# Recommendations for the Neurophysiological Assessment of Conditioned Pain Modulation: A Systematic Review of Nociceptive Blink Reflex and Nociceptive Withdrawal Reflex Protocols

**DOI:** 10.1002/ejp.70149

**Published:** 2025-10-27

**Authors:** Josh Murphy, Sophie Clarke, Paul Strutton, Celia Morgan, Kirsty Bannister, Sam W. Hughes

**Affiliations:** ^1^ Department of Clinical and Biomedical Sciences, Medical School Building University of Exeter Exeter UK; ^2^ Brain and Research and Imaging Centre, School of Psychology University of Plymouth Plymouth UK; ^3^ The Nick Davey Laboratory, Department of Surgery and Cancer, Faculty of Medicine Imperial College London London UK; ^4^ Department of Psychology University of Exeter Exeter UK; ^5^ Department of Life Sciences, South Kensington Imperial College London London UK

## Abstract

**Background and Objective:**

The nociceptive blink reflex (NBR) and nociceptive withdrawal reflex (NWR) are increasingly used as neurophysiological test stimuli to assess conditioned pain modulation (CPM). However, methodological inconsistencies limit reproducibility and cross‐study comparison. This systematic review aimed to evaluate experimental protocols and outcome measures used in CPM studies employing NBR or NWR, and to propose recommendations for standardisation.

**Databases and Data Treatment:**

Following PRISMA guidelines, a systematic search of PubMed, Cochrane and Ovid databases was conducted. Studies were included if they assessed CPM in healthy adults using NBR or NWR. Data were extracted on stimulation parameters, electrode configuration, participant positioning, data acquisition, signal processing and CPM protocols. Sixteen studies met inclusion criteria (6 NBR, 10 NWR).

**Results:**

Across studies, stimulation protocols varied in frequency, pulse duration and threshold calibration. Electrode placements and participant positioning were often inconsistently reported. Data acquisition methods differed in sampling rates and filtering, and analysis windows were not always defined. CPM protocols included cold pressor, heat and electrocutaneous stimulation. Outcome measures included both amplitude‐ and threshold‐based metrics.

**Conclusions:**

This review highlights significant methodological heterogeneity in reflex‐based CPM research in healthy participants. Standardisation of stimulation parameters, recording techniques, and CPM protocols is needed to enhance comparability and reproducibility across studies. To address this, we provide structured recommendations, based on GRADE classifications, for standardising protocols related to experimental setup, data acquisition and CPM outcome measures for both the NBR and NWR.

**Significance Statement:**

Reflex‐based conditioned pain modulation demonstrates methodological inconsistencies, highlighting the need for standardised protocols. With standardised protocols recommended in this review, future efforts should focus on enhancing its reliability, reproducibility and clinical applicability.

## 1 Introduction

The application of a heterotopic noxious conditioning stimulus can be used to activate a spinally projecting monoaminergic inhibitory mechanism within the descending pain modulation system (DPMS), referred to as diffuse noxious inhibitory control (DNIC) in animals (Le Bars et al. 1979) and conditioned pain modulation (CPM) in humans (Yarnitsky 2010). Neurophysiological test stimuli, such as the lower limb nociceptive withdrawal reflex (NWR) and nociceptive blink reflex (NBR), have been used in place of traditional psychophysical test stimuli in CPM experiments (Kennedy et al. 2016). The NBR and NWR provide distinct insights into CPM mechanisms and reflect the top‐down modulation of nociceptive signals at the level of the brainstem and dorsal horn of the spinal cord, respectively. Critically, this approach has the potential to improve reliability and be used as objective biomarkers during the development of new centrally acting pain therapies (Tracey et al. 2019).

The choice of whether to use the NBR or NWR in neurophysiological CPM protocols is dependent on the circuitry of interest. The NBR is a polysynaptic, pontomedullary reflex within the brainstem reticular formation (Kofler et al. 2024). The reflex is elicited using electrical stimulation using either concentric nociceptive specific electrodes that preferentially activate A‐delta fibres, or patch electrodes which can activate both innocuous A‐beta and A‐delta fibres within the supraorbital nerve which manifest as R1 and R2 responses, respectively (Kaube et al. 2000). After supraorbital nerve activation, afferent signals travel via the trigeminal nerve to the pons and medulla, reaching the spinal trigeminal nucleus (STN) (Marin et al. 2015). Within the STN, wide dynamic range (WDR) neurons integrate noxious and innocuous inputs and project via interneurons to the facial motor nucleus (FMN) in the pons. Motor neurons in the FMN then activate the facial nerve (cranial nerve VII) (Jerath and Kimura 2019), which activates the orbicularis oculi muscles, producing a blink.

The NWR represents a broader protective response to noxious stimuli in the muscles of the lower limb. High‐intensity stimulation (1.5–2× threshold) of the plantar arch or sural nerve preferentially activates A‐delta fibres, which transmit nociceptive signals to the dorsal horn. This type of stimulation will not avoid co‐activation of A‐beta fibres; however, the distinct early (20–40 ms) and late (90–150 ms) components of the reflex allow the nociceptive response to be measured without exclusively activating A‐delta fibres. In the dorsal horn, the nociceptive signals synapse with projection neurons (i.e., WDR neurons) to relay nociceptive signals to the brain. Excitatory interneurons in the dorsal and ventral horns also initiate a withdrawal reflex, activating proximal (e.g., biceps femoris) and distal (e.g., tibialis anterior) muscles (Andersen 2007; Sandrini et al. 2005).

These distinct mechanistic pathways can provide key insights into DPMS function and dysfunction when used alongside heterotopic noxious conditioning stimuli as part of CPM paradigms. However, the current lack of standardisation in experimental setup, data acquisition and protocols used to elicit the NBR or NWR makes it difficult to determine optimal protocols, compare CPM outcomes and replicate findings across studies. In this review, we systematically evaluate methodologies used to evoke, measure and modulate the NBR and NWR and identify key areas for standardisation for use as test stimuli within neurophysiological CPM protocols.

## 2 Methods

This systematic review was performed using the Preferred Reporting Items for Systematic Reviews and Meta‐Analyses (PRISMA) guidelines. The review was pre‐registered with PROSPERO 2024 CRD42024538831. A deviation from the protocol was made in that screening and data extraction were conducted by a single reviewer. All other aspects of the protocol were followed as planned.

Available from https://www.crd.york.ac.uk/PROSPERO/view/CRD42024538831.

### 2.1 Search Strategy: NBR

The primary search was conducted using PubMed, Cochrane and Ovid. We used the search terms ‘[Nociceptive blink reflex OR R2 blink reflex AND healthy]’ whilst limiting to humans in Ovid. We used the search terms ‘[((((nociceptive blink reflex) OR (R2 blink reflex)) AND (healthy)) AND (human)) AND (modulation)]’ in PubMed. We used the search terms ‘[Nociceptive blink reflex OR R2 blink reflex AND modulation AND healthy]’ in Cochrane. Duplicates were removed.

**FIGURE 1:**
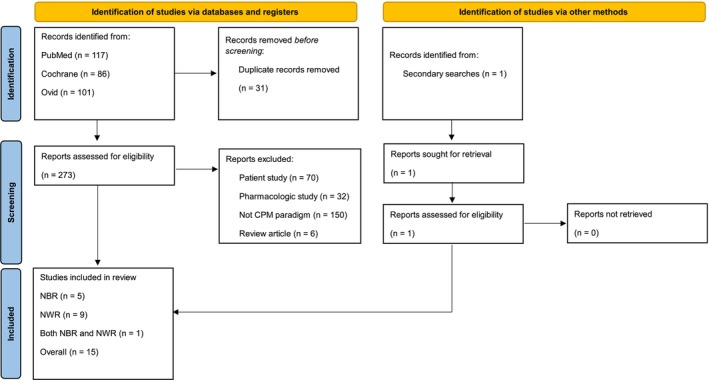
PRISMA flowchart. The initial search was performed across PubMed, Cochrane and Ovid databases. Duplicate entries were excluded (*n* = 30), and the remaining full‐text articles were assessed based on established inclusion and exclusion criteria. The reports which met these criteria (*n* = 15) were selected for data extraction and subsequent analysis. An additional study (*n* = 1) was later incorporated manually. This resulted in a total of 16 reports included in the final analysis.

This assessment was completed by screening each article against the specified criteria, which was completed by J.M. After this primary search, a secondary search was conducted to ensure no articles were missed with the terms ‘[(nociceptive blink reflex) OR (R2 blink reflex) OR (nociceptive blink) AND (human)]’. This search identified one additional study that fits our criteria and was therefore included for further analysis. No other modified search terms or manual searches identified further studies that met the inclusion criteria. The results of these searches and the numbers included are outlined in Figure 1. Searches were limited to the English language since the most recent update of the databases on 7 November 2024.

### 2.2 Study Eligibility: NBR

The NBR results (*n* = 101) were assessed based on predefined inclusion and exclusion criteria. Studies were included if they primarily focused on CPM in healthy human participants, with a particular emphasis on the R2 component of the NBR. Studies were excluded if they investigated models other than CPM, patient cohorts to ensure the review is focused on the basic mechanisms of the BR, pharmacological interventions, pain treatments, or if they were review articles.

### 2.3 Search Strategy: NWR

The primary search was conducted using PubMed, Cochrane and Ovid. We used the search terms ‘[Nociceptive withdrawal reflex OR nociceptive flexion reflex AND healthy AND modulation]’ whilst limiting to humans in Ovid. We used the search terms ‘[((((nociceptive withdrawal reflex) OR (nociceptive flexion reflex)) AND (healthy)) AND (human)) AND (modulation)]’ whilst limiting nociceptive withdrawal reflex and nociceptive flexion reflex to Title/Abstract in PubMed. We used the search terms ‘[Nociceptive withdrawal reflex OR nociceptive flexion reflex AND human AND modulation AND healthy]’ whilst limiting nociceptive withdrawal reflex and nociceptive flexion reflex to ‘Title Abstract keyword’ in Cochrane. Duplicates were removed.

This assessment was completed by screening each article against the specified criteria, which was completed by J.M. No other modified search terms or manual searches identified further studies that met the inclusion criteria. The results of these searches and numbers included are outlined in Figure 1. Searches were limited to the English language, since the most recent update of the databases on 7 November 2024.

### 2.4 Study Eligibility: NWR

The results (*n* = 172) were assessed based on predefined inclusion and exclusion criteria. Studies were included if they primarily focused on CPM in healthy human participants, with a particular emphasis on the NWR in the lower limb. Studies were excluded if they investigated models other than CPM, patient cohorts to ensure the review is focused on the basic mechanisms of the NWR, pharmacological interventions, pain treatments, or if they were review articles (Figure 1).

### 2.5 Data Extraction

For all studies included in the final analysis, data extraction was conducted by J.M., capturing key methodological details. Extracted variables included sample size, sampling rate, bandwidth, band‐pass filters, gain, software used, sweep length, latency of response, stimulation type, recording and stimulating electrodes used, electrode placement, participant position, stimulation intensity (mA), pain threshold (mA), analysis type, study design, conditioning stimulus used, number of male and female participants and participant age.

The risk of bias for all identified studies was evaluated using the Cochrane risk of bias assessment tool (Higgins et al. 2011), with modifications to suit the analysis of CPM studies. Two authors (J.M. and S.C.) independently assessed the risk of bias for each study, examining all five domains outlined by the tool. Any discrepancies between assessments were discussed and resolved through consensus. The final risk of bias evaluations were summarised and reported for each domain.

### 2.6 Data Synthesis

A systematic narrative synthesis was conducted to evaluate CPM effects on the BR and NWR, ensuring that included studies measured at least one baseline reflex response followed by a post‐conditioning measurement. Key methodological aspects were analysed, including: (1) Experimental setup; (2) Reflex data acquisition and signal processing; and (3) CPM protocols and outcome measures. The synthesis provides an overview of current approaches to neurophysiological CPM assessment and identifies suggested areas for standardisation.

### 2.7 Development of Recommendations

In addition to synthesising the available evidence, we formulated practical recommendations for experimental set‐up when measuring reflex outcomes. Recommendations were derived through a structured consensus process within the review team, informed by: (i) patterns observed in the included studies, (ii) methodological considerations from related fields, (iii) feasibility and safety considerations, and (iv) expert consensus (Guyatt et al. 2011).

We graded each recommendation according to the GRADE approach (Tables 1 and 2). The certainty of evidence supporting each recommendation was rated as high, moderate, low, or very low, depending on risk of bias, consistency, directness, precision and publication bias in the underlying studies. The strength of recommendation (strong vs. conditional) was based on the balance between benefits and risks, quality of supporting evidence and practical considerations. Where direct evidence was lacking, recommendations were classified as expert consensus and graded as conditional, very low certainty.

**TABLE 1:** Experimental setup parameters for the nociceptive blink reflex. Breakdown of the experimental setup parameters described in Sections 3.2.2 and 3.2.3.

Author	*N*	Stimulation electrode	Recording electrode	Sampling rate	Bandwidth	Band‐pass filters	Latencies	Stimulation frequency and pulse duration	Trials	ISI	Participant position
Kinukawa et al. (2021)	15 (13 NBR)	Supraorbital foramen (IES), eyebrow (TS), concentric bipolar needle electrode	EMG/EP measuring system, orbicularis oculi muscle	10 kHz	Not reported	10–2000 Hz	27–97 ms	200 Hz, 2.0 ms (IES), 0.2 ms (TS)	6	13–17 s	Seated upright, eyes open
Drummond et al. (2018)	20	Custom‐built concentric, supraorbital region	Modified disposable Cleartrode Surface, orbicularis oculi muscle	Not reported	Not reported	Not reported	27–87 ms	200 Hz, 0.5 ms	10	15 s	Not reported
Rehberg et al. (2012)	50 (41 NBR)	Planar concentric, supraorbital foramen	Surface, orbicularis oculi muscle	Not reported	Not reported	2 Hz‐1 kHz	27–87 ms	200 Hz, 0.5 ms	18	18–22 s	Seated upright, eyes closed
Jürgens et al. (2008)	34	specific electrode by selective A‐d stimulation, supraorbital groove	Surface, orbicularis oculi muscle	Not reported	50 Hz to 2.5 kHz	Not reported	30–90 ms	200 ms	6	5 s	Not reported
Giffin et al. (2004)	28 (19 for CPM)	Concentric surface, forehead	Silver chloride, orbicularis oculi muscle	2.5 kHz	1 Hz‐1 kHz	Not reported	27–87 ms	Not reported	6	15–17 s	Not reported
Ellrich and Treede (1998)	11	Supraorbital foramen	Surface, mid lower eyelid	Not reported	Not reported	50–1600 Hz	27–87 ms	200 ms	9	10 s	Laid supine, eyes lightly closed.

**TABLE 2:** CPM protocols and outcome measures for the nociceptive blink reflex. Overview of the thresholds, analysis type and conditioning stimulus used as described in Section 3.2.4.

Author	Stimulation intensity	Analysis type	Conditioning stimulus
Kinukawa et al. (2021)	1.5× threshold	EMG AUC	Cold water immersion of the hand at 10°C
Drummond et al. (2018)	Not reported	EMG AUC	Heat on the forearm
Rehberg et al. (2012)	The stimulation intensities used for the nociceptive blink reflex were 8.8 ± 0.4, 14.5 ± 0.6, and 22.1 ± 0.9 mA (mean ± SE)	EMG AUC	Cold water immersion 12°C
Jürgens et al. (2008)	Not reported	EMG AUC	GON activation
Giffin et al. (2004)	1.5× threshold	EMG AUC	Cold water immersion of the hand at 0°C
Ellrich and Treede (1998)	Reflex thresholds were the minimum intensities evoking a response that could be discriminated from baseline	EMG AUC	Heat on the forearm

## 3 Results

The primary search conducted using databases provided 304 results. These results were processed by removing duplicates (*n* = 30), leaving 274 publications to be reviewed. Each publication was then assessed to determine whether it met the inclusion and exclusion criteria. After this, 15 studies were identified. After modifying search terms, a further 1 publication was identified. This meant a final list of 16 studies was taken forward to data extraction. Search results are outlined in Figure 1.

### 3.1 Risk of Bias Assessment

For all included studies, two authors (J.M. and S.C.) independently assessed the risk of bias using the Cochrane tool for assessing risk of bias (Higgins et al. 2011). The risk of bias assessment criteria was adapted for suitability for CPM studies (e.g., blinding of a participant to the condition isn't possible in CPM studies). It was assessed based on six standardised domains: selection bias, performance bias, detection bias, attrition bias, reporting bias and other biases. Selection bias was evaluated based on the type of study. For interventional studies, this involved assessing random sequence generation and allocation concealment, whereas for observational studies, the presence of a control condition or group was examined. Performance bias focused on the blinding of participants and personnel; if blinding practices were not explicitly stated, no blinding was assumed, and the study was rated as having a high risk of bias. Detection bias, which considered the blinding of outcome assessments, was rated similarly if blinding was not mentioned. Attrition bias was assessed by examining incomplete data sets and the reasons for missing data, such as participant dropout. Reporting bias was evaluated by looking for evidence of selective reporting and determining whether the study directly assessed pre‐specified outcome measures. If specific details were not provided and no significant omissions were evident, the study was rated as unclear risk. Other biases, such as sample size adequacy and demographic representation in terms of age and sex, were also considered. This comprehensive approach ensured a systematic evaluation of potential biases across all included studies.

**FIGURE 2:**
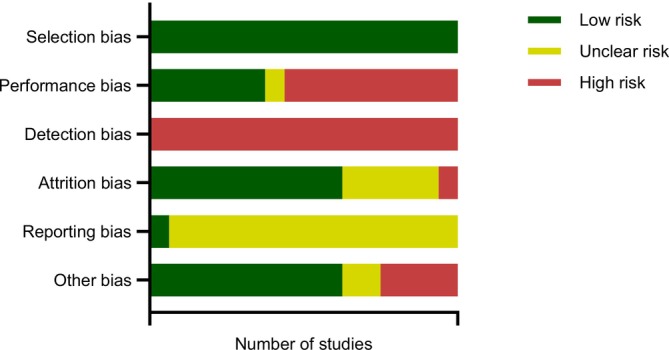
Risk of bias assessment for all included studies. For each study, the risk of bias was assessed based on six standardised domains: Selection bias, performance bias, detection bias, attrition bias, reporting bias and other biases. Assessment criteria was adapted for suitability for CPM studies. Green indicates the number showing low risk, yellow for an unclear risk and red for high risk. This includes all studies identified (*n* = 16).

Our assessment found that selection bias was a consistent strength across all included studies, with 100% of the studies demonstrating a low risk of bias. This was attributed to the presence of clear control or baseline conditions in each study. However, performance and detection bias emerged as the primary concerns. Most studies did not provide evidence of participant blinding, and none of the studies explicitly reported blinding of outcome assessment, resulting in a high risk of bias in these areas. For attrition, reporting and other biases, the majority of assessments were rated as either low or unclear risk, with minimal concerns identified. Within the category of ‘other bias’, the most notable issue was the limited sample sizes or an uneven distribution of male and female participants, which could impact the generalisability of the findings (Figure 2). For a more in‐depth view of the risk of bias see Table S1.

### 3.2 Nociceptive Blink Reflex

#### 3.2.1 Study Population

The studies investigating the NBR had varied sample sizes and age ranges. Kinukawa et al. (2021) included 15 male participants initially, although two were excluded due to issues eliciting the reflex, with a mean age of 37.1 ± 10.8 years. Drummond et al. (2018) conducted their research on 20 participants, evenly divided by gender, ranging in age from 20 to 51 years with a mean of 26.3 years (SD = 7.5). Rehberg et al. (2012) started with 50 subjects split into 25 males and 25 females, with 41 specifically analysed for NBR after excluding 7 participants due to incomplete data, averaging 26 ± 6 years. Jürgens et al. (2008) studied 34 participants, equally balanced in gender distribution, aged between 18 and 37 years with a mean age of 25.1 ± 4.15 years. Giffin et al. (2004) recruited 28 participants, with 19 specifically tested for DNIC/CPM, consisting of 13 females and 15 males, aged 21–33 years. Ellrich and Treede (1998) recruited 11 participants consisting of 4 males and 7 females with an age range of 20–28 years. The total sample size across all studies was 158 with 140 participants completing the NBR and an overall mean participant age of 28 years. The total sample size by sex was 86 males and 72 females, although the proportion that completed the NBR is unclear (Table 3).

**TABLE 3:** Recommendations for standardising nociceptive blink reflex (NBR) protocols. Summaries of recommended methodological standards for eliciting and recording the Nociceptive Blink Reflex (NBR) in experimental settings. Key areas include stimulation parameters, electrode configuration, participant positioning, trial repetition, data acquisition, signal processing and CPM protocol design. GRADE classifications for each recommendation have also been provided following guidelines set out in Section 2.6.

Area	Suggestions for standardisation of the NBR	GRADE classification
Experimental set up	Stimulating electrode type and placements should be standardised across participants (e.g., concentric bipolar electrodes placed 10 mm above supraorbital foramen).	Strong, high
	Participant positioning should be consistent (e.g., seated upright, eyes open).	Conditional, very low
	Stimulation parameters clearly defined and consistent across studies (e.g., double‐ or triple‐pulse trains at 0.5 ms pulse width and 5 ms inter‐pulse intervals).	Strong, moderate
	Minimum of 5 averaged trials per condition, and 8–15 s inter‐stimulus intervals (randomised).	Strong, moderate
	Recording electrode type and placements should be standardised across participants (e.g., self‐adhesive surface electrodes placed on the orbicularis oculi muscle, directly below the eye and at the outer corner).	Strong, high
	Ground electrode type and placement should be standardised across participants (e.g., self‐adhesive surface electrodes placed on the ipsilateral inside of the nose bridge).	Conditional, very low
Data acquisition and signal processing	Stadardised sampling rates (e.g., 2 kHz) and filter settings (e.g., band‐pass 10 Hz–1 kHz).	Conditional, moderate
	EMG data should be consistently processed by rectifying the signal.	Strong, high
	Defined analysis window for the R2 component (e.g., 27–87 ms post‐stimulation onset).	Strong, high
CPM protocols and outcome measures	Conditioning stimuli should be standardised across participants (e.g., cold water immersion at fixed temperature and duration).	Strong, high
	Changes in EMG area under the curve (AUC) should be used for evaluating reflex amplitude changes post‐conditioning.	Strong, high

#### 3.2.2 Experimental Setup

##### 3.2.2.1 Stimulation Parameters

Stimulation frequencies and intensities varied widely among studies. Kinukawa et al. (2021) employed high‐frequency stimulation at 200 Hz, with pulse durations of 2.0 ms for intradermal electrical stimulation (IES) and 0.2 ms for transcutaneous stimulation (TS). Drummond et al. (2018) and Rehberg et al. (2012) used pulse trains of three monopolar square‐wave pulses, each lasting 0.5 ms, separated by 5 ms inter‐pulse intervals (i.e., 200 Hz). Jürgens et al. (2008) applied monopolar square‐wave pulses lasting 200 ms. Giffin et al. (2004) did not report their stimulation frequency or pulse duration. Ellrich and Treede (1998) used square wave pulses with a duration of 200 ms and did not report their pulse duration. Specific intensities based on thresholds were not consistently reported across all studies. We recommend double‐ or triple‐pulse trains at 0.5 ms pulse width and 5 ms inter‐pulse intervals (GRADE: strong, moderate).

##### 3.2.2.2 Electrode Configuration

Recording and stimulating electrodes, along with their placements, were similar across studies. Kinukawa et al. (2021) recorded from the left orbicularis oculi muscle, with stimulation electrodes positioned superior to the right supraorbital foramen (IES) and around the right eyebrow (TS). Drummond et al. (2018) employed disposable electrodes to record from the orbicularis oculi muscle, applying custom‐built concentric stimulating electrodes bilaterally on the supraorbital region. Rehberg et al. (2012) recorded from surface electrodes placed infraorbitally and at the root of the nose, with concentric electrodes positioned approximately 10 mm above the left supraorbital foramen. Giffin et al. (2004) also recorded infraorbitally and at the nose root using bilateral silver chloride electrodes, with concentric stimulation electrodes placed on the forehead. Ellrich and Treede (1998) recorded infraorbitally with the active electrode over the mid lower eyelid; they placed the reference on the temple and a ground at the middle forehead. Stimulation electrodes were placed at the left supraorbital nerve. Jürgens et al. (2008) stimulated using a specific electrode by selective A‐delta stimulation. Recording electrode details were not explicitly described by Jürgens et al. (2008), however, refer to Busch et al. (2006) for their setup using surface electrodes placed infraorbitally below the eyes for recording. We recommend bilateral recording at the obicularis oculi muscle with surface electrodes and stimulation with concentric bipolar electrodes placed 10 mm above the supraorbital foramen (GRADE: strong, high). We suggest ground electrode placement at the ipsilateral inside of the nose bridge (GRADE: conditional, very low).

##### 3.2.2.3 Participant Positioning

Participant positioning varied substantially. Kinukawa et al. (2021) seated participants upright, while Rehberg et al. (2012) positioned participants in a quiet room, seated in a flexible chair with 120‐degree hip flexion with eyes closed. Ellrich and Treede (1998) had participants lie supine on a bed with eyes closed. However, participant positioning details were not reported by Drummond et al. (2018), Jürgens et al. (2008) or Giffin et al. (2004). We suggest participants be seated upright with eyes open (GRADE: conditional, very low).

##### 3.2.2.4 Trial Repetition and Intervals

Across the reviewed NBR studies, reflex measurements were typically averaged over multiple repetitions to enhance signal reliability. Most protocols used 5–10 trials per condition (Kinukawa et al. 2021; Drummond et al. 2018; Jürgens et al. 2008; Giffin et al. 2004; Ellrich and Treede 1998), with Rehberg et al. (2012) employing up to 18 trials for greater consistency in detecting changes during CPM. The inter‐stimulus interval (ISI) between trials ranged from 5 to 17 s, with some studies using fixed intervals (Drummond et al. 2018; Jürgens et al. 2008; Ellrich and Treede 1998) and others employing randomised intervals (Kinukawa et al. 2021; Rehberg et al. 2012; Giffin et al. 2004) to reduce participant anticipation and habituation. These practices support recommendations for averaging NBR responses over at least 5 trials, with 8–15 s ISIs, ideally randomised within this range (GRADE: strong, moderate).

#### 3.2.3 Data Acquisition and Signal Processing

##### 3.2.3.1 Recording Parameters

Sampling rates and filter settings varied across studies. Kinukawa et al. (2021) reported a high sampling rate of 10 kHz and used a band‐pass filter ranging from 10 Hz to 2 kHz. Rehberg et al. (2012) applied band‐pass filters between 2 Hz and 1 kHz but did not report the sampling rate. Giffin et al. (2004) employed a sampling rate of 2.5 kHz and a bandwidth of 1 Hz–1 kHz, though further filtering details were not specified. Ellrich and Treede (1998) reported bandpass filters of 50–1600 Hz; however, they did not report other parameters. Drummond et al. (2018) and Jürgens et al. (2008) did not clearly report their filtering or sampling parameters. We suggest sampling rates of 2 kHz and band‐pass filtering at 10 Hz–1 kHz (GRADE: conditional, moderate).

##### 3.2.3.2 Signal Processing and Analysis Window

Signal processing varied but typically included rectification and averaging of EMG traces (Kinukawa et al. 2021; Rehberg et al. 2012; Jürgens et al. 2008). Reflex analysis focused on the R2 component, with analysis windows most commonly defined between 27–87 ms post‐stimulation onset (Kinukawa et al. 2021; Drummond et al. 2018; Giffin et al. 2004; Ellrich and Treede 1998). One study extended the upper limit to 97 ms (Rehberg et al. 2012). Jürgens et al. (2008) referenced Busch et al. (2006) who used a window of 30–90 ms. The R1 component was less frequently analysed, being primarily non‐nociceptive (Ellrich and Treede 1998). We recommend EMG data be consistently processed by rectifying the signal (GRADE: strong, high) and an analysis window of 27–87 ms post‐stimulation onset (GRADE: strong, high).

#### 3.2.4 CPM Protocols and Outcome Measures

##### 3.2.4.1 Conditioning Stimuli

CPM protocols used various conditioning stimuli (Table 2). Kinukawa et al. (2021) utilised cold‐water immersion at 10°C. Rehberg et al. (2012) applied cold water at 12°C. Giffin et al. (2004) used cold water immersion at 0°C. Drummond et al. (2018) and Ellrich and Treede (1998) used heat stimuli on the forearm. Jürgens et al. (2008) applied electrical stimulation of the greater occipital nerve. We recommend that the conditioning stimulus be standardised across participants, for example, cold water immersion at a fixed temperature and duration (GRADE: strong, high).

##### 3.2.4.2 Outcome Metrics

Most studies used Area Under the Curve (AUC) of the R2 component as the primary outcome measure. Giffin et al. (2004) also referenced changes in pain thresholds. Not all studies detailed whether amplitude or threshold modulation was the intended measure, though AUC has been widely adopted (Tables 2 and 3). We recommend using changes in EMG AUC to evaluate NBR amplitude changes post‐conditioning (GRADE: strong, high).

### 3.3 Nociceptive Withdrawal Reflex

#### 3.3.1 Study Population

Studies examining the NWR varied in terms of sample size and participant age. Guekos et al. (2023) included 16 participants evenly split between males and females, averaging 26.8 ± 4.7 years. Lie et al. (2019) involved 25 participants, with 14 males and 11 females aged between 18 and 45 years. Schliessbach et al. (2019) had a larger sample, initially involving 146 subjects, with NWR successfully elicited in 73 participants, reporting an average age of 42.5 ± 17.4 years. Jure et al. (2019) studied 16 participants (9 males, 7 females) aged 20–35 years, whereas Biurrun Manresa et al. (2014) recruited 39 males (34 included in the final analysis) ranging from 18 to 65 years. Jurth et al. (2014) included 40 participants split into 20 males and 20 females; however, only 36 completed the study. It is unclear what their age range was. Lewis et al. (2012) recruited 19 male participants with a mean age of 31 ± 5 years. For the NWR section of their protocol, Rehberg et al. (2012) had a slightly larger sample size of 43; it is unclear what the sex of participants was for the NWR; however, the initial sample had an even split of 25 males and 25 females. The age of participants was 26 ± 6 SD. Serrao et al. (2004) recruited 36 participants with 20 females and 16 males. The age range was 24–39 years. Willer et al. (1984) had a smaller sample size of 10 participants, 8 male and 2 female. The age range was 26–44 years. Across all studies, a total of 397 participants were recruited, with 308 completing the NWR section of the study. The overall mean age of participants was 29.1 years. Gender distribution showed a predominance of male participants, with 224 males and 173 females taking part in the studies; however, the proportion that completed the NWR is unclear (Table 4).

**TABLE 4:** Experimental setup parameters for the nociceptive withdrawal reflex. Categorisation of the experimental setup parameters described in Sections 3.3.2 and 3.3.3.

Author	*N*	Stimulation electrode	Recording electrode	Sampling rate	Bandwidth	Band‐pass filters	Latencies	Stimulation frequency and pulse duration	Trials	ISI	Participant position
Guekos et al. (2023)	16	Sural nerve—two surface, MP—one of foot sole, one on Dorsum	Surface electrodes	48 kHz, downsampled to 6 kHz	Not reported	10 Hz to 500 Hz	60–150 ms	200 Hz, 1 ms	8	5–15 s, randomised	Semi‐supine, knees bent
Lie et al. (2019)	25	Silver chloride surface	Surface electrodes	Not reported	Not reported	Not reported	70–150 ms	200 Hz, 1 ms	10	8–12 s, randomised	Semi‐supine, knees bent
Schliessbach et al. (2019)	146 (73 NWR)	Bipolar surface	Surface electrodes	Not reported	Not reported	Not reported	50–150 ms	1 ms	Thresholding	Not applicable as tested NWR threshold	Supine
Jure et al. (2019)	16	Self‐adhesive cathode and large anode.	Surface electrodes	2400 Hz	Not reported	5–450 Hz	60–250 ms	200 Hz, 1 ms	10	8–10 s	Supine
Biurrun Manresa et al. (2014)	39 (34 NWR)	Bipolar silver chloride surface	Silver chloride electrodes	Not reported	Not reported	Not reported	60–180 ms	1 ms	Thresholding	Not applicable as tested NWR threshold	Supine
Jurth et al. (2014)	40 (36 NWR)	Surface	Surface electrodes	Not reported	Not reported	2 Hz to 1 kHz	90–180 ms	200 Hz, 1 ms	20	8–12 s, randomised	Semi‐supine, knees bent
Lewis et al. (2012)	19	Bipolar	Surface electrodes	5 kHz	Not reported	10–1 kHz	85–150 ms	250 Hz, 1 ms	10	8–12 s	Stood, leg hanging
Rehberg et al. (2012)	50 (43 NWR)	Two silver chloride surface	Silver chloride electrodes	Not reported	Not reported	2 Hz to 1 kHz	90–150 ms	1 ms	36	8–12 s, randomised	Semi‐supine, knees bent
Serrao et al. (2004)	36	Surface	Surface electrodes	Not reported	Not reported	Not reported	90–130 ms	1 ms	20	5–20 s, randomised	Semi‐supine, knees bent
Willer et al. (1984)	10	Surface	Surface electrodes	Not reported	Not reported	Not reported	80–180 ms	1 ms	Unclear	Not reported	Semi‐supine, knees bent

#### 3.3.2 Experimental Setup

##### 3.3.2.1 Stimulation Parameters

Stimulation was typically delivered using a constant current stimulator as a train of five rectangular pulses, each 1 ms in duration, at 200 Hz, perceived as a single stimulus (Rehberg et al. 2012; Guekos et al. 2023; Lie et al. 2019; Schliessbach et al. 2019; Jure et al. 2019; Biurrun Manresa et al. 2014; Jurth et al. 2014; Lewis et al. 2012; Serrao et al. 2004). Willer et al. (1984) used a burst of eight pulses, and all studies used either 200 or 250 Hz burst frequency. Inter‐stimulus intervals were generally randomised between 5 and 12 s. Stimulation intensity was based on individual thresholds in most studies found using the staircase method or NRS ratings. Guekos et al. (2023) used 1, 1.5, and 2× threshold. Schliessbach et al. (2019) based it on a reflex amplitude exceeding 20 μV for at least 10 ms. Lewis et al. (2012) used 120% of the threshold. Willer et al. (1984) used a subjective visual scale. Serrao et al. (2004) used 1.2× threshold. Jure et al. (2019) reported mean thresholds of 8.6 ± 4.5 mA (range 3–18 mA), while Biurrun Manresa et al. (2014) reported mean thresholds of approximately 17 mA. Lie et al. (2019) used fixed intensities (6–8 mA). Rehberg et al. (2012) used 9.2, 14.9 and 21.8 mA as three levels of intensity. Jurth et al. (2014) did not report their threshold. We recommend use of a standard stimulation protocol of five 1 ms rectangular pulses at 200 Hz (GRADE: strong, high) and stimulation intensities clearly related to individual threshold calculations, for example, 1.5× the NWR threshold intensity (GRADE: strong, high).

##### 3.3.2.2 Electrode Configuration

All studies except for Lie et al. (2019) recorded from the biceps femoris (BF) muscle. Lie et al. (2019) recorded from the tibialis anterior (TA) muscle. As well as the BF and TA, Jure et al. (2019) and Guekos et al. (2023) recorded from the rectus femoris (RF) muscle, whereas Jure et al. (2019) also included the soleus (SOL) muscle. Surface electrodes positioned approximately 20 mm apart in alignment with SENIAM guidelines (Stegeman and Hermens 2007) were used in all studies. Stimulating electrodes targeted the medial plantar nerve (Guekos et al. 2023; Lie et al. 2019; Jure et al. 2019; Lewis et al. 2012) or sural nerve (Rehberg et al. 2012; Guekos et al. 2023; Schliessbach et al. 2019; Biurrun Manresa et al. 2014; Jurth et al. 2014; Serrao et al. 2004; Willer et al. 1984) typically placed on the medial arch or dorsum of the foot, or just posterior to the lateral malleolus respectively. Electrode configurations included monopolar and bipolar. We recommend consistent placement of stimulation electrodes either at the plantar arch of the foot or the sural nerve and recording electrodes at the BF or TA (GRADE: strong, high).

##### 3.3.2.3 Participant Positioning

Participants were generally tested in semi‐supine or supine postures on medical beds or plinths, often in quiet rooms. Guekos et al. (2023), Lie et al. (2019), Jurth et al. (2014), Rehberg et al. (2012), Serrao et al. (2004) and Willer et al. (1984) used a variation of semi‐supine posture with knees bent; Jure et al. (2019), Schliessbach et al. (2019) and Biurrun Manresa et al. (2014) all reported comfortable reclined postures. Lewis et al. (2012) had participants stand with their legs hanging passively. We recommend participants be semi‐supine with knees bent and supported at a consistent angle (GRADE: strong, moderate).

##### 3.3.2.4 Repetition and ISI

Studies ranged from 5–36 trials per condition to ensure response reliability apart from Schliessbach et al. (2019) and Biurrun Manresa et al. (2014) which used NWR threshold rather than repeated trials. Willer et al. (1984) did not report their number of trials clearly. Inter‐stimulus intervals ranged from 5–20 s to avoid habituation and participant anticipation, and most used randomisation (e.g., 8–12 s range). This was not applicable for Schliessbach et al. (2019) and Biurrun Manresa et al. (2014) as they used the NWR threshold and Willer et al. (1984) did not report their intervals clearly. We recommend a minimum of 8–12 averaged trials per condition and randomised inter‐stimulus intervals of 10–15 s (GRADE: strong, high).

#### 3.3.3 Data Acquisition and Signal Processing

##### 3.3.3.1 Recording Parameters

Sampling rates varied across studies. Guekos et al. (2023) used a 48 kHz sampling rate, which was later down‐sampled to 6 kHz; Jure et al. (2019) used 2.4 kHz, Lewis et al. (2012) used 5 kHz and all other studies failed to report their sampling rate. Filters used were 5–500 Hz (Jure et al. 2019), 10–500 Hz (Guekos et al. 2023), 2 Hz to 1 kHz (Rehberg et al. 2012; Jurth et al. 2014) or 10 Hz to 1 kHz (Lewis et al. 2012). All other studies failed to report their filtering methods. We suggest standardised sampling rates of 2–2.5 kHz and band‐pass filtering of 10 Hz to 1 kHz (GRADE: conditional, low).

##### 3.3.3.2 Signal Processing and Analysis Window

Signal processing commonly included rectification, followed by AUC integration or RMS transformation. For amplitude‐based analysis, Guekos et al. (2023), Rehberg et al. (2012) and Serrao et al. (2004) used AUC. Jure et al. (2019) used muscle synergy and latency‐based analysis. Lewis et al. (2012) applied RMS amplitude. Threshold‐based approaches were used by Lie et al. (2019), Schliessbach et al. (2019), Biurrun Manresa et al. (2014) and Willer et al. (Willer et al. 1984). Jurth et al. (2014) only reported their ICCs. Analysis windows for reflex latency were most commonly set in the range of 50–180 ms post‐stimulation onset. We recommend consistent rectification of EMG signals (GRADE: strong, high) and a defined reflex analysis window of 60–150 ms post‐stimulation onset (GRADE: strong, high).

#### 3.3.4 CPM Protocols and Outcome Measures

##### 3.3.4.1 Conditioning Stimuli

CPM protocols typically involved thermal conditioning (Table 5). Guekos et al. (2023) applied heat stimulation to the foot or forearm. Schliessbach et al. (2019), Rehberg et al. (2012), Jure et al. (2019), Lewis et al. (2012), Serrao et al. (2004), Lie et al. (2019) and Biurrun Manresa et al. (2014) used the cold pressor test, with immersion temperatures standardised (e.g., 1.5°C–12°C) for 60–90 s. Jurth et al. (2014) and Willer et al. (1984) used hot water immersion, although Willer et al. (1984) also used cold water, muscular exercise of the hand under ischaemia and painful pinch to the nasal septum. Consistency of the conditioning protocol was maintained for comparability across sessions. We recommend that the conditioning stimulus be standardised across participants; for example, cold water immersion at a fixed temperature and duration (GRADE: strong, high).

**TABLE 5:** CPM protocols and outcome measures for the nociceptive withdrawal reflex. This table provides an overview of the thresholds, analysis type and conditioning stimulus used as described in Section 3.3.4.

Author	Stimulation intensity	Analysis type	Conditioning stimulus
Guekos et al. (2023)	1, 1.5 and 2× threshold	Cohen's *d* and AUC	Heat in lower leg and foot sole—32°C, 36°C, 39°C, 42°C, 45°C, and 46°C
Lie et al. (2019)	Peak *z*‐score of 12	NWR threshold change in mA	Cold pressor—7°C
Schliessbach et al. (2019)	Reflex amplitude exceeding 20 μV for at least 10 ms	Threshold	Cold pressor—1.5°C ± 1°C
Jure et al. (2019)	2× threshold intensity	Muscle synergy analysis and latencies	Cold pressor—2.7°C ± 0.5°C
Biurrun Manresa et al. (2014)	1.5× threshold intensity	Threshold	Cold pressor—below 2°C
Jurth et al. (2014)	Not reported	ICCs for mean z‐scores	Hot water—46.5°C
Lewis et al. (2012)	120% of threshold intensity	RMS amplitude	Cold pressor—12°C ± 1°C
			Ischemic arm—250 mmHg followed by 20 wrist extensions with 2.5 kg weight.
Rehberg et al. (2012)	30, 50, 70 NRS	AUC	Cold pressor—12°C
Serrao et al. (2004)	1.2× threshold intensity	Threshold and AUC	Cold pressor—2°C–4°C
Willer et al. (1984)	Subjective scores on visual scale	Threshold	Hot water—40,42, 44, 45, 46, 47, 47.5°C
			Cold water—6°C
			Ischemic arm—1.5× systolic blood pressure then 10 watts with left forearm
			Nasal septum pinch—4.5 kg/cm²

##### 3.3.4.2 Outcome Metrics

Outcome measures varied according to the study hypothesis. Amplitude‐based metrics were commonly used to assess reflex magnitude changes using AUC (Rehberg et al. 2012; Guekos et al. 2023; Serrao et al. 2004), RMS (Lewis et al. 2012), or muscle synergy metrics (Jure et al. 2019). Threshold‐based modulation (e.g., mA change) was reported in Biurrun Manresa et al. (2014), Serrao et al. (2004), Schliessbach et al. (2019), Lie et al. (2019) and Willer et al. (1984). Jurth et al. (2014) used ICCs for change in reflex size using mean *z*‐scores. Although AUC is sometimes used and aligns with NBR methodologies, it is not universally applied in NWR studies. Therefore, future studies would benefit from standardising the choice of modulation metric, with AUC recommended for consistency and sensitivity to inhibitory changes during CPM (Tables 5 and 6). We suggest uniform evaluation of amplitude changes through AUC to quantify reflex modulation (GRADE: conditional, very low) and clear and consistent reporting of either amplitude‐ or threshold‐based measures for reflex modulation across studies (GRADE: conditional, low).

**TABLE 6:** Recommendations for standardising nociceptive withdrawal reflex (NWR) protocols. Standardisation and classification for NWR studies assessing CPM. It includes best practices for stimulation protocols, muscle and nerve electrode placements, participant positioning, inter‐trial timing, recording parameters and appropriate outcome measures. GRADE classifications for each recommendation have also been provided following guidelines set out in Section 2.6.

Area	Suggestions for standardisation of the NWR	GRADE classification
Experimental set up	Consistent stimulating and recording electrode placement (e.g., plantar arch of the foot and sural nerve, biceps femoris and tibialis anterior muscles).	Strong, high.
	Standard participant positioning (semi‐supine, knees bent at consistent angle).	Strong, moderate.
	Use of a standard stimulation protocol (e.g., five 1 ms rectangular pulses at 200 Hz).	Strong, high.
	Standard stimulation intensities clearly related to individual thresholds (e.g., 1.5× individual NWR threshold intensity or stimulus response functions).	Strong, high.
	Minimum of 8–12 averaged trials per condition and inter‐stimulus intervals of 10–15 s (randomised).	Strong, high.
Data acquisition and signal processing	Standardised sampling rate (e.g., 2–2.5 kHz) and band‐pass filtering (e.g., 10Hz—1 kHz).	Conditional, low.
	Consistent signal processing steps including rectification of EMG data.	Strong, high.
	Defined analysis window for NWR latency (e.g., consistent measurement window of 80–150 ms post‐stimulation onset).	Strong, high.
CPM protocols and outcome measures	Standard conditioning stimuli (e.g., cold pressor at fixed temperature and duration)	Strong, high.
	Uniform evaluation of amplitude changes through AUC to quantify reflex modulation.	Conditional, very low.
	Clear reporting and consistent use of either amplitude‐based and threshold‐based measures for reflex modulation across studies.	Conditional, low.

## 4 Discussion

This systematic review identified inconsistencies in protocols and outcome measures used in NBR and NWR studies assessing CPM. Across 15 included studies (9 NWR, 5 NBR, 1 joint NWR and NBR), there were differences in the reporting of many of the acquisition parameters used, including the stimulation parameters, sampling rates, and filtering practices. For outcome measures, most studies employed amplitude‐based reflex measures, often using AUC or RMS amplitude, though some also used threshold‐based approaches. Notably, the choice between amplitude and threshold modulation was rarely justified based on predefined or mechanistic hypotheses. CPM protocols ranged from cold pressor and heat stimulation to electrocutaneous conditioning, with differences in the reporting of stimulus intensity and outcome metrics. Together, these methodological disparities highlight substantial heterogeneity that limits reproducibility and comparability across studies. Using this information, alongside expert opinion, we developed new GRADE recommendations to guide standardisation of NBR and NWR protocols in neurophysiological CPM paradigms.

Based on the GRADE recommendations outlined in this review, future NBR studies should adopt a standardised experimental setup encompassing participant positioning (upright, eyes open, minimising distraction), consistent electrode placement on the orbicularis oculi and supraorbital region and standardised stimulation parameters (double/triple‐pulse trains, 0.5 ms width, 5 ms inter‐pulse intervals). At least five averaged trials with randomised inter‐stimulus intervals of 8–15 s, data acquisition at a 2 kHz sampling rate with 10–1000 Hz band‐pass filtering, and analysis of rectified responses within the 27–87 ms window are recommended. Standardisation of conditioning stimulus intensity and duration is also essential. Although some recommendations were rated as conditional/low due to inconsistent reporting, such standardisation would substantially improve comparability and reproducibility of brainstem CPM research.

Compared with the nociceptive blink reflex, there is greater methodological consensus for the NWR, reflecting its more frequent use in the literature (Kennedy et al. 2016; Ramaswamy and Wodehouse 2021). Consequently, many of our recommendations are rated as strong and high in our GRADE recommendations. To facilitate standardisation, we advise semi‐supine participant positioning with consistent knee flexion, electrode placement on the tibialis anterior or biceps femoris, and stimulation of the plantar arch or sural nerve. Stimulation should be delivered at 1.5 times reflex threshold using trains of five rectangular pulses (1 ms duration, 200 Hz). A minimum of 8–12 trials with randomised inter‐stimulus intervals of 10–15 s, data acquisition at 2–2.5 kHz sampling rate with 10–1000 Hz filtering, and analysis of rectified responses in the 80–150 ms window are recommended. Clear reporting of threshold changes and reflex amplitude (via AUC) should be prioritised, with consistent conditioning stimuli across studies. Adopting these parameters will enhance reproducibility and comparability of NWR and CPM research across laboratories.

A critical methodological question revolves around whether the chosen reflex measure is designed to capture amplitude modulation or threshold changes. Amplitude‐based protocols typically apply suprathreshold stimuli so that the reflex is reliably evoked (Palmieri et al. 2004), highlighting changes in the central excitability of the reflex arc, particularly when inhibitory or facilitatory mechanisms are triggered by a conditioning stimulus (Ellrich and Treede 1998). By contrast, threshold‐based approaches incrementally increase stimulus intensity until a reflex is just elicited, thereby assessing how a conditioning stimulus shifts the minimal current required for reflex onset (Rehberg et al. 2012). Although threshold‐based protocols may be better tolerated by participants, they can be influenced by peripheral conduction velocity, skin impedance, and local tissue properties (Tankisi et al. 2020), thus reflecting both descending inhibition at the central synapse and peripheral excitability changes. Consequently, it is possible that threshold measures may be more susceptible to confounding peripheral factors outside the central reflex arc. Once a reflex is reliably triggered (i.e., using suprathreshold stimulation), amplitude changes more directly indicate central excitability modulation, as peripheral influences play a smaller role in shaping the final response amplitude (Islam et al. 2021). Therefore, selecting threshold or amplitude endpoints should be guided by the hypothesised site(s) of modulation: if central (e.g., dorsal horn, trigeminal nucleus) changes are of particular interest, amplitude‐based methods may be more specific, whereas threshold‐based methods provide a broader view that may also capture peripheral adaptations (Leone et al. 2021; Ydrefors et al. 2020). For the NBR this is reflected in the consistent choice of amplitude‐based AUC to uncover central brainstem mechanisms. For the NWR clarification is more critical due to a variation of threshold‐ and amplitude‐based choices. Measuring both would be the clearest way to display results.

Building on the distinction between threshold‐ and amplitude‐based methods, the latter typically involves rectifying the EMG signal to capture absolute muscle activity (Lewis et al. 2015). Once rectified, researchers must decide whether to compute the root mean square RMS amplitude or integrate the entire response to obtain the AUC. While RMS amplitude provides a moment‐to‐moment measure suited to stable or monophasic bursts (Fukuda et al. 2010; Ogalo et al. 2024), reflex responses often display multiple peaks or latency variations, particularly under inhibitory influences (Jure et al. 2019). In such cases, AUC, by capturing both amplitude and duration, can offer greater sensitivity to partial suppressions or prolonged reflex components (Taniguchi et al. 2022). For the NBR, there is clear consensus that AUC is the most reliable measurement. The NWR is divided, with only three studies employing AUC; others used RMS, latencies or threshold‐based methods, which arguably do not reflect the whole reflex. Thus, amplitude‐based measures calculated over a defined window (e.g., 27–87 ms for the NBR, 80–150 ms for the NWR) can robustly quantify changes attributable to a conditioning stimulus. Physiologically, a reduction in amplitude (whether via RMS or AUC) indicates more effective descending inhibition within the trigeminal nucleus (NBR) or dorsal horn (NWR) (Jure et al. 2019; Watson and Drummond 2014).

A wide variety of conditioning stimuli ranging from cold water immersion (Rehberg et al. 2012; Giffin et al. 2004; Schliessbach et al. 2019) to heat (Drummond et al. 2018; Lie et al. 2019), electrical nerve activation (Jürgens et al. 2008), or ischemic exercise (Biurrun Manresa et al. 2014) can all successfully engage descending inhibitory pathways for neurophysiological CPM protocols. This implies that the modality does not influence CPM effects as each approach can likely trigger the necessary modulatory mechanisms. However, modality differences complicate cross‐study comparisons, as stimulus parameters (temperature, location, duration) need careful standardisation to ensure consistent levels of nociceptive input (Damien et al. 2018). Additionally, the duration of a conditioning stimulus for the cold pressor is often 60–90 s since varying intervals or repeated cycles may introduce adaptation effects rather than purely reflecting descending inhibition (Lewis et al. 2012).

Few studies reported stimulus–response graphs, yet these are vital for a comprehensive understanding of how CPM modulates reflexes across varying stimulus intensities. Such graphs allow researchers to see whether changes in reflex amplitude occur uniformly or only at specific points along the intensity range (Iyer and Madhavan 2019). Plotting response amplitude at incremental stimulus intensities can reveal whether inhibition is broad‐based, which would be evident as a downward shift across the entire amplitude–intensity function, or more selective, such as a threshold shift without significantly altering suprathreshold amplitudes. If there is a broad downward shift in amplitudes across all intensities, then calculating an average amplitude across all intensities can provide a convenient single metric to gauge overall reflex excitability (Yarnitsky 2010; Willer et al. 1984). Consequently, stimulus–response curves clarify whether modulation is uniform or intensity‐dependent in the broader context of the reflex's response profile (Tansley et al. 2019).

Although we are confident in the recommendations presented in this paper, it is important to acknowledge key limitations of our review. First, both study screening and data extraction were performed by a single reviewer, which increases the risk of errors and subjective bias. This may have led to the inadvertent exclusion of relevant studies or inaccuracies in data capture. Although resource constraints sometimes necessitate single‐reviewer processes, best practice is dual independent screening and extraction to maximise reliability and reduce bias. In our review, a dual process was only feasible for the risk of bias assessment. Second, GRADE classifications are influenced by several factors, and in cases where reporting was limited or study findings were inconsistent, some recommendations relied more heavily on expert judgement. While this remains consistent with GRADE methodology, it resulted in some recommendations being graded as conditional and of very low certainty. Nonetheless, these recommendations provide a foundation on which future studies can build. Finally, methodological variability in CPM studies extends beyond the test stimulus and includes factors such as the type and intensity of the conditioning stimulus, the timing between conditioning and test stimuli, habituation effects, and the relative intensities of the stimuli. Not all these factors are consistently reported or controlled across studies, and some, such as cold‐water immersion conditioning, are inherently difficult to standardise due to individual differences in pain perception and tolerance. While these aspects can substantially influence CPM outcomes, they were not comprehensively addressed in the present review. These procedural differences should also be kept in mind when interpreting results.

In summary, the present systematic review highlights extensive methodological heterogeneity in reflex‐based CPM studies using the NBR or NWR. To advance this field, we propose a set of standardised guidelines encompassing participant positioning, data acquisition, signal processing, and consistent CPM outcome measures. We also emphasise the importance of clarifying whether amplitude or threshold changes represent the primary endpoint. By specifying standard experimental procedures and carefully aligning measurement strategies with experimental hypotheses, future studies can strengthen both the reproducibility and clinical relevance of reflex‐based CPM research.

## Author Contributions

This systematic review was designed by J.M., S.C., and S.W.H. The data were collected by J.M. The risk of bias was carried out by J.M. and S.C. The data synthesis and results were produced by J.M. and S.W.H. The manuscript was edited by J.M., K.B., P.S., C.M., and S.W.H. All authors have approved the final version of the manuscript and agree to be accountable for all aspects of the work.

## Conflicts of Interest

The authors declare no conflicts of interest.

## Supporting information


**Table S1:** Per‐study risk of bias.

## References

[ejp70149-bib-0001] Andersen, O. K. 2007. “Studies of the Organization of the Human Nociceptive Withdrawal Reflex: Focus on Sensory Convergence and Stimulation Site Dependency.” Acta Physiologica 189: 1–35. 17439638 10.1111/j.1748-1716.2007.01706.x

[ejp70149-bib-0002] Biurrun Manresa, J. A. , R. Fritsche , P. H. Vuilleumier , et al. 2014. “Is the Conditioned Pain Modulation Paradigm Reliable? A Test‐Retest Assessment Using the Nociceptive Withdrawal Reflex.” PLoS One 9, no. 6: e100241. 24950186 10.1371/journal.pone.0100241 PMC4065000

[ejp70149-bib-0003] Busch, V. , W. Jakob , T. Juergens , W. Schulte‐Mattler , H. Kaube , and A. May . 2006. “Functional Connectivity Between Trigeminal and Occipital Nerves Revealed by Occipital Nerve Blockade and Nociceptive Blink Reflexes.” Cephalalgia 26, no. 1: 50–55. 16396666 10.1111/j.1468-2982.2005.00992.x

[ejp70149-bib-0004] Damien, J. , L. Colloca , C.‐É. Bellei‐Rodriguez , et al. 2018. “Pain Modulation: From Conditioned Pain Modulation to Placebo and Nocebo Effects in Experimental and Clinical Pain.” International Review of Neurobiology 139: 255–296. 30146050 10.1016/bs.irn.2018.07.024 PMC6175288

[ejp70149-bib-0005] Drummond, P. D. , A. Bell , and L. Vo . 2018. “Painful Stimulation of a Sensitized Site in the Forearm Inhibits Ipsilateral Trigeminal Nociceptive Blink Reflexes.” Experimental Brain Research 236, no. 7: 2097–2105. 29754196 10.1007/s00221-018-5255-x

[ejp70149-bib-0006] Ellrich, J. , and R.‐D. Treede . 1998. “Characterization of Blink Reflex Interneurons by Activation of Diffuse Noxious Inhibitory Controls in Man.” Brain Research 803, no. 1–2: 161–168. 9729360 10.1016/s0006-8993(98)00646-5

[ejp70149-bib-0007] Fukuda, T. Y. , J. O. Echeimberg , J. E. Pompeu , et al. 2010. “Root Mean Square Value of the Electromyographic Signal in the Isometric Torque of the Quadriceps, Hamstrings and Brachial Biceps Muscles in Female Subjects.” Journal of Applied Research 10, no. 1: 32–39.

[ejp70149-bib-0008] Giffin, N. , Z. Katsarava , A. Pfundstein , J. Ellrich , and H. Kaube . 2004. “The Effect of Multiple Stimuli on the Modulation of the ‘Nociceptive’ Blink Reflex.” Pain 108, no. 1–2: 124–128. 15109515 10.1016/j.pain.2003.12.014

[ejp70149-bib-0009] Guekos, A. , A. C. Grata , M. Hubli , M. Schubert , and P. Schweinhardt . 2023. “Are Changes in Nociceptive Withdrawal Reflex Magnitude a Viable Central Sensitization Proxy? Implications of a Replication Attempt.” Clinical Neurophysiology 145: 139–150. 36272950 10.1016/j.clinph.2022.09.011

[ejp70149-bib-0010] Guyatt, G. H. , A. D. Oxman , H. J. Schünemann , P. Tugwell , and A. Knottnerus . 2011. “GRADE Guidelines: A New Series of Articles in the Journal of Clinical Epidemiology.” Journal of Clinical Epidemiology 64, no. 4: 380–382. 21185693 10.1016/j.jclinepi.2010.09.011

[ejp70149-bib-0011] Higgins, J. P. , D. G. Altman , P. C. Gøtzsche , et al. 2011. “The Cochrane Collaboration's Tool for Assessing Risk of Bias in Randomised Trials.” BMJ 343: d5928. 22008217 10.1136/bmj.d5928 PMC3196245

[ejp70149-bib-0012] Islam, M. A. , T. S. Pulverenti , and M. Knikou . 2021. “Neuronal Actions of Transspinal Stimulation on Locomotor Networks and Reflex Excitability During Walking in Humans With and Without Spinal Cord Injury.” Frontiers in Human Neuroscience 15: 620414. 33679347 10.3389/fnhum.2021.620414 PMC7930001

[ejp70149-bib-0013] Iyer, P. C. , and S. Madhavan . 2019. “Characterization of Stimulus Response Curves Obtained With Transcranial Magnetic Stimulation From Bilateral Tibialis Anterior Muscles Post Stroke.” Neuroscience Letters 713: 134530. 31585209 10.1016/j.neulet.2019.134530 PMC7226675

[ejp70149-bib-0014] Jerath, N. , and J. Kimura . 2019. “F Wave, A Wave, H Reflex, and Blink Reflex.” Handbook of Clinical Neurology 160: 225–239. 31277850 10.1016/B978-0-444-64032-1.00015-1

[ejp70149-bib-0015] Jure, F. A. , F. G. Arguissain , J. A. B. Manresa , and O. K. Andersen . 2019. “Conditioned Pain Modulation Affects the Withdrawal Reflex Pattern to Nociceptive Stimulation in Humans.” Neuroscience 408: 259–271. 30999033 10.1016/j.neuroscience.2019.04.016

[ejp70149-bib-0016] Jürgens, T. , V. Busch , O. Opatz , W. Schulte‐Mattler , and A. May . 2008. “Low‐Frequency Short‐Time Nociceptive Stimulation of the Greater Occipital Nerve Does Not Modulate the Trigeminal System.” Cephalalgia 28, no. 8: 842–846. 18513262 10.1111/j.1468-2982.2008.01612.x

[ejp70149-bib-0017] Jurth, C. , B. Rehberg , and F. von Dincklage . 2014. “Reliability of Subjective Pain Ratings and Nociceptive Flexion Reflex Responses as Measures of Conditioned Pain Modulation.” Pain Research & Management 19, no. 2: 93–96. 24555177 10.1155/2014/698246 PMC4028659

[ejp70149-bib-0018] Kaube, H. , Z. Katsarava , T. Käufer , H.‐C. Diener , and J. Ellrich . 2000. “A New Method to Increase Nociception Specificity of the Human Blink Reflex.” Clinical Neurophysiology 111, no. 3: 413–416. 10699400 10.1016/s1388-2457(99)00295-3

[ejp70149-bib-0019] Kennedy, D. L. , H. I. Kemp , D. Ridout , D. Yarnitsky , and A. S. Rice . 2016. “Reliability of Conditioned Pain Modulation: A Systematic Review.” Pain 157, no. 11: 2410–2419. 27559835 10.1097/j.pain.0000000000000689 PMC5228613

[ejp70149-bib-0020] Kinukawa, T. A. , K. Inui , T. Taniguchi , et al. 2021. “Conditioned Pain Modulation: Comparison of the Effects on Nociceptive and Non‐Nociceptive Blink Reflex.” Neuroscience 468: 168–175. 34147564 10.1016/j.neuroscience.2021.06.019

[ejp70149-bib-0021] Kofler, M. , M. Hallett , G. D. Iannetti , et al. 2024. “The Blink Reflex and Its Modulation–Part 1: Physiological Mechanisms.” Clinical Neurophysiology 160: 130–152. 38102022 10.1016/j.clinph.2023.11.015 PMC10978309

[ejp70149-bib-0022] Le Bars, D. , A. H. Dickenson , and J.‐M. Besson . 1979. “Diffuse Noxious Inhibitory Controls (DNIC). I. Effects on Dorsal Horn Convergent Neurones in the Rat.” Pain 6, no. 3: 283–304. 460935 10.1016/0304-3959(79)90049-6

[ejp70149-bib-0023] Leone, C. , A. Di Lionardo , G. Di Pietro , et al. 2021. “How Different Experimental Models of Secondary Hyperalgesia Change the Nociceptive Flexion Reflex.” Clinical Neurophysiology 132, no. 12: 2989–2995. 34715423 10.1016/j.clinph.2021.08.018

[ejp70149-bib-0024] Lewis, G. N. , A. Leys , D. A. Rice , and P. J. McNair . 2015. “Subconscious Manipulation of Pain Expectation Can Modulate Cortical Nociceptive Processing.” Pain Practice 15, no. 2: 117–123. 24325269 10.1111/papr.12157

[ejp70149-bib-0025] Lewis, G. N. , H. Luke , D. A. Rice , K. Rome , and P. J. McNair . 2012. “Reliability of the Conditioned Pain Modulation Paradigm to Assess Endogenous Inhibitory Pain Pathways.” Pain Research & Management 17, no. 2: 98–102. 22518372 10.1155/2012/610561 PMC3393056

[ejp70149-bib-0026] Lie, M. U. , E. Petriu , D. Matre , et al. 2019. “Psychophysical or Spinal Reflex Measures When Assessing Conditioned Pain Modulation?” European Journal of Pain 23, no. 10: 1879–1889. 31359580 10.1002/ejp.1462

[ejp70149-bib-0027] Marin, J. C. , A. R. Gantenbein , K. Paemeleire , H. Kaube , and P. J. Goadsby . 2015. “Nociception‐Specific Blink Reflex: Pharmacology in Healthy Volunteers.” Journal of Headache and Pain 16, no. 1: 87. 26449227 10.1186/s10194-015-0568-7 PMC4598330

[ejp70149-bib-0028] Ogalo, E. , L. D. Linde , H. Ro , O. Ortiz , J. L. Kramer , and M. J. Berger . 2024. “Evaluating Peripheral Neuromuscular Function With Brief Movement‐Evoked Pain.” Journal of Neurophysiology 131, no. 5: 789–796. 38353653 10.1152/jn.00472.2023 PMC11383610

[ejp70149-bib-0029] Palmieri, R. M. , C. D. Ingersoll , and M. A. Hoffman . 2004. “The Hoffmann Reflex: Methodologic Considerations and Applications for Use in Sports Medicine and Athletic Training Research.” Journal of Athletic Training 39, no. 3: 268. 16558683 PMC522151

[ejp70149-bib-0030] Ramaswamy, S. , and T. Wodehouse . 2021. “Conditioned Pain Modulation—A Comprehensive Review.” Neurophysiologie Clinique 51, no. 3: 197–208. 33334645 10.1016/j.neucli.2020.11.002

[ejp70149-bib-0031] Rehberg, B. , J. H. Baars , J. Kotsch , P. Koppe , and F. von Dincklage . 2012. “Comparison of Trigeminal and Spinal Modulation of Pain and Nociception.” International Journal of Neuroscience 122, no. 6: 298–304. 22225522 10.3109/00207454.2011.649868

[ejp70149-bib-0032] Sandrini, G. , M. Serrao , P. Rossi , A. Romaniello , G. Cruccu , and J. C. Willer . 2005. “The Lower Limb Flexion Reflex in Humans.” Progress in Neurobiology 77, no. 6: 353–395. 16386347 10.1016/j.pneurobio.2005.11.003

[ejp70149-bib-0033] Schliessbach, J. , C. Lütolf , K. Streitberger , P. Scaramozzino , L. Arendt‐Nielsen , and M. Curatolo . 2019. “Reference Values of Conditioned Pain Modulation.” Scandinavian Journal of Pain 19, no. 2: 279–286. 30699074 10.1515/sjpain-2018-0356

[ejp70149-bib-0034] Serrao, M. , P. Rossi , G. Sandrini , et al. 2004. “Effects of Diffuse Noxious Inhibitory Controls on Temporal Summation of the RIII Reflex in Humans.” Pain 112, no. 3: 353–360. 15561391 10.1016/j.pain.2004.09.018

[ejp70149-bib-0035] Stegeman, D. , and H. Hermens . 2007. “Standards for Surface Electromyography: The European Project Surface EMG for Non‐Invasive Assessment of Muscles (SENIAM).” Enschede: Roessingh Research and Development 10, no. 8: 108–112.

[ejp70149-bib-0036] Taniguchi, T. , T. A. Kinukawa , N. Takeuchi , et al. 2022. “A Minimally Invasive Method for Observing Wind‐Up of Flexion Reflex in Humans: Comparison of Electrical and Magnetic Stimulation.” Frontiers in Neuroscience 16: 837340. 35281508 10.3389/fnins.2022.837340 PMC8904398

[ejp70149-bib-0037] Tankisi, H. , D. Burke , L. Cui , et al. 2020. “Standards of Instrumentation of EMG.” Clinical Neurophysiology 131, no. 1: 243–258. 31761717 10.1016/j.clinph.2019.07.025

[ejp70149-bib-0038] Tansley, S. N. , L. C. Macintyre , L. Diamond , et al. 2019. “Conditioned Pain Modulation in Rodents Can Feature Hyperalgesia or Hypoalgesia Depending on Test Stimulus Intensity.” Pain 160, no. 4: 784–792. 30681982 10.1097/j.pain.0000000000001454

[ejp70149-bib-0039] Tracey, I. , C. J. Woolf , and N. A. Andrews . 2019. “Composite Pain Biomarker Signatures for Objective Assessment and Effective Treatment.” Neuron 101, no. 5: 783–800. 30844399 10.1016/j.neuron.2019.02.019 PMC6800055

[ejp70149-bib-0040] Watson, D. H. , and P. D. Drummond . 2014. “Cervical Referral of Head Pain in Migraineurs: Effects on the Nociceptive Blink Reflex.” Headache: The Journal of Head and Face Pain 54, no. 6: 1035–1045. 10.1111/head.12336 24666216

[ejp70149-bib-0041] Willer, J. , A. Roby , and D. L. Bars . 1984. “Psychophysical and Electrophysiological Approaches to the Pain‐Relieving Effects of Heterotopic Nociceptive Stimuli.” Brain 107, no. 4: 1095–1112. 6509310 10.1093/brain/107.4.1095

[ejp70149-bib-0042] Yarnitsky, D. 2010. “Conditioned Pain Modulation (the Diffuse Noxious Inhibitory Control‐Like Effect): Its Relevance for Acute and Chronic Pain States.” Current Opinion in Anesthesiology 23, no. 5: 611–615. 20543676 10.1097/ACO.0b013e32833c348b

[ejp70149-bib-0043] Ydrefors, J. , T. Karlsson , U. Wentzel Olausson , et al. 2020. “Automated Nociceptive Withdrawal Reflex Measurements Reveal Normal Reflex Thresholds and Augmented Pain Ratings in Patients With Fibromyalgia.” Journal of Clinical Medicine 9, no. 6: 1992. 32630430 10.3390/jcm9061992 PMC7356211

