# Supplementary material for: Recommendations for the Neurophysiological Assessment of Conditioned Pain Modulation: A Systematic Review of Nociceptive Blink Reflex and Nociceptive Withdrawal Reflex Protocols
Source: Eur J Pain. 2025 Oct 27;29(10):e70149. doi: 10.1002/ejp.70149 (PMC12558665; doi:10.1002/ejp.70149)
Supplement: Supplementary file 1 — Table S1: Per‐study risk of bias. [file EJP-29-0-s001.docx]

| **Study** | **Selection bias** | **Performance bias** | **Detection bias** | **Attrition bias** | **Reporting bias** | **Other bias** |
| --- | --- | --- | --- | --- | --- | --- |
| Kinukawa et al. |  |  |  |  |  |  |
| Drummond et al. |  |  |  |  |  |  |
| Rehberg et al. |  |  |  |  |  |  |
| Jurgens et al. |  |  |  |  |  |  |
| Giffin et al. |  |  |  |  |  |  |
| Ellrich and Treede |  |  |  |  |  |  |
| Guekos et al. |  |  |  |  |  |  |
| Lie et al. |  |  |  |  |  |  |
| Schliessbach et al. |  |  |  |  |  |  |
| Jure et al. |  |  |  |  |  |  |
| Biurrun Manresa et |  |  |  |  |  |  |
| Jurth et al. |  |  |  |  |  |  |
| Lewis et al. |  |  |  |  |  |  |
| Serrao et al. |  |  |  |  |  |  |
| Willer et al. |  |  |  |  |  |  |
|  |  |  |  |  |  |  |
| Key: |  |  |  |  |  |  |
| **Low** |  |  |  |  |  |  |
| **Unclear** |  |  |  |  |  |  |
| **High** |  |  |  |  |  |  |

**Supplementary table 1. Per-study risk of bias.** Study by study a risk of bias carried out for all NBR and NWR studies according to 6 categories: selection bias, performance bias, detection bias, attrition bias, reporting bias and other bias. This is explained fully in section ‘3.1 Risk of bias assessment’. A rating for each paper in each category has been given corresponding to either green – low risk, yellow – unclear risk or red – high risk.
